# Liquid Crystal-Based Optical Biosensor for Quantitative, Highly Sensitive Detection of Proteins

**DOI:** 10.3390/bios16030168

**Published:** 2026-03-17

**Authors:** Lorenzo Fiorentini, Raouf Barboza, Maria Logovatovskaya, Elia Rocchetti, Paolo Mariani, Liana Lucchetti

**Affiliations:** 1 Dipartimento di Scienze e Ingegneria della Materia, dell’Ambiente ed Urbanistica, Università Politecnica delle Marche, Via Brecce Bianche, 60131 Ancona, Italy; l.fiorentini@staff.unipm.it (L.F.); e.rocchetti@staff.univpm.it (E.R.); 2 Dipartimento di Scienze della Vita e dell’Ambiente, Università Politecnica delle Marche, Via Brecce Bianche, 60131 Ancona, Italy; p.mariani@staff.univpm.it

**Keywords:** nematic liquid crystals, optical biosensor, protein detection

## Abstract

We report a highly sensitive label-free optical biosensor based on nematic liquid crystals, for the detection of proteins. The principles of biosensing are based on the change in the liquid crystal alignment induced by biomolecules adsorbed on the cell inner surface, which can be easily detected with a polarizing optical microscope. Although this approach is well-known, we propose here an experimental strategy that allows us to reach a detection limit of the order of 10^−13^ g/mL, orders of magnitude higher than the one reported in the literature for similar biosensors. Furthermore, our method leads to assessing a well-defined, specific dependence of protein concentration on cell birefringence, for rapid quantitative biosensing. The proposed biosensor can additionally be used for the detection of antibodies.

## 1. Introduction

**Figure 1:**
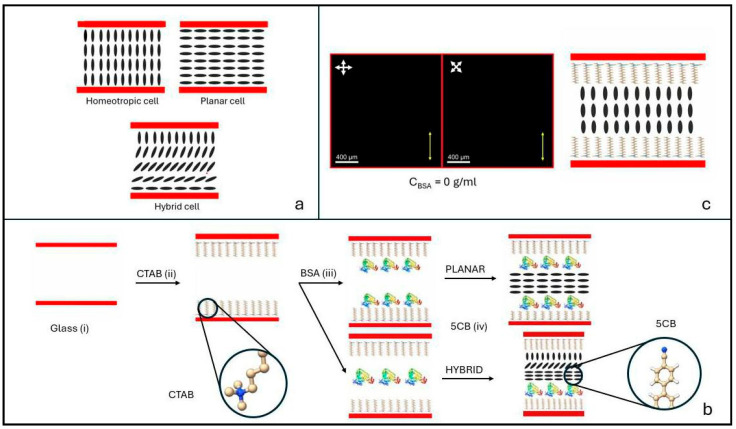
(**a**) Most common LC cell alignments: homeotropic, planar and hybrid. Red bars are the glass substrates; black rods represent the LC molecules, which average orientation defines the LC optic axis. (**b**) Sketch of the different steps leading to the realization of the optical biosensor: (i) bare glass substrates, (ii) glass substrates coated by CTAB, (iii) single or double coating of BSA molecules deposited by dip coating on the CTAB-treated surfaces, and (iv) final cell filled with 5CB. Illustrations are not to scale. (**c**) POM image of a reference cell obtained with no BSA. White crossed arrows represent the crossed POM polarizers. The yellow arrow indicates the direction of 5CB flow during cell filling. The cell is optically isotropic and appears dark regardless of its orientation with respect to the polarizers’ axis, indicating homeotropic alignment of the LC molecules.

A nematic liquid crystal (NLC) is a fluid of structurally anisotropic molecules exhibiting long-range orientational order [1]. The direction of the mean molecular orientation is expressed by the nematic director, a unit vector indicated as **n**. When a drop of NLC is placed between two properly treated glass substrates, it is possible to induce a preferred alignment of **n** at the surfaces. Thanks to the long-range correlation of the liquid crystal molecular order, the surface orientation is then transferred to the bulk. In this way, a sort of soft single crystal can be easily obtained, i.e., a liquid crystal sample where the molecular orientation is uniform over distances much larger than the molecular scale [2]. The three most common configurations obtained by this method are classified as homeotropic, planar and hybrid alignments, and are sketched in Figure 1a. In homeotropic samples the liquid crystal director **n** is normal to the confining surfaces, homogeneous planar alignment characterizes samples where the average molecular orientation is parallel to the surfaces along a well-defined direction, and hybrid alignment is a distorted configuration generated by homeotropic orientation on one surface and planar orientation on the other.

In NLC the director **n** also defines the optic axis. The long-range orientational order makes these materials anisotropic so that the refractive indexes for light polarized along or perpendicular to the optic axis are different, a property known as birefringence [2]. How an NLC sample looks under a crossed polarizer thus depends on the average molecular alignment. Specifically, homeotropic samples are optically isotropic and appear dark, while planar and hybrid cells affect the polarization of the incident light and a certain amount of it passes through the microscope analyzer [2]. When the coupling between NLC molecules and the aligning surface is somehow disturbed, the distribution of the director field **n** changes and this produces a variation in the cell birefringence, which is easily detectable under a polarized optical microscope (POM). This optical amplification is the basis of most liquid crystal optical biosensors [3,4,5,6,7].

In the simplest version, the starting configuration consists of an NLC cell with homeotropic alignment [8]. In these samples the confining surfaces are glass slabs coated with the proper surfactant, generally Dimethyloctadecyl[3-(trimethoxysilyl)propyl]ammonium chloride (DMOAP). The dark appearance of homeotropic samples under crossed polarizers guarantees the maximum possible contrast in the case of alignment disturbance. Indeed, as a biomolecule is attached to one of the confining surfaces, the alignment changes locally and the cell acquires a certain amount of birefringence, which is visualized as a certain amount of light passing through the POM analyzer. Although in principle, the contrast offered by non-homeotropic areas in a homeotropic background is high and due to NLC elasticity an area larger than the surface region affected contributes to the observed birefringence, the detection limit reached by this method is typically not very high, being of the order of 10^−8^ g/mL (e.g., 0.15 nM for a protein such as Bovine Serum Albumin—BSA—with a molecular weight of about 66.5 kDa) [9,10]. Several strategies have been proposed to improve this value. A field-assisted signal amplification was recently reported, consisting of applying an electric field near the Freedericksz threshold to a homeotropically aligned NLC with negative dielectric anisotropy [11]. The role of the field is that of destabilizing the initial alignment, thus facilitating the action of the target analyte. A different approach was based on the measurement of the electric capacitance of liquid crystal cells as a function of the concentration of the detected protein [12]. In both papers the detection limit is lower than 10^−9^ g/mL; however, POM images lose resolution at low concentration of the target analyte, and the light transmitted from the analyzer in these conditions is hardly distinguishable from impurities in the original cell. Using a bent-core NLC with a low bend elastic constant, a biosensing strategy based on the flexoelectric effect was recently described, reporting a sensitivity of the order of 10^−10^ g/mL for BSA [13].

In this work we use a different strategy based on sample preparation, which allows us to obtain images with a good contrast down to a 10^−13^ g/mL analyte concentration (1.5 fM for BSA). Although better detection limits can be reached in biosensors based on different techniques [14,15], this is the lowest value reported for optical biosensors based on systems as simple as conventional liquid crystal cells. It is noteworthy that the same very remarkable detection limit was reached a few years ago using an optical biosensor based on wavelength-shift in the resonance of liquid crystal whispering gallery mode resonators [16], which are beautiful systems but significantly more complicated.

Our approach enables us to assess a correspondence between the analyte concentration and the cell birefringence, thus realizing a biosensor in which the visual signal provides information on both the presence of the analyte and its concentration. The biosensor we propose reacts in a different way to different proteins, which indicates a certain degree of specificity. Additional tests demonstrated the possibility of labeled detection of polyclonal antibodies.

## 2. Materials and Methods

We used the nematic liquid crystal 4-Cyano-4′-pentylbiphenyl (5CB, Nematel, Mainz, Germany) as a sensing mesogen and the model protein Bovine Serum Albumin (BSA, Thermo Fisher Scientific, Waltham, MA, USA) as a target analyte. BSA is a globular protein derived from bovine blood serum widely used in biochemical and biophysical research due to its stability, availability, and well-characterized structure [17]. BSA has a molecular weight of approximately 66.5 kDa and consists of 583 amino acids arranged in a single polypeptide chain. At physiological pH, BSA carries a net negative charge, with an isoelectric point around 4.7. The surfactant selected as both aligning agent and biorecognition element was the cationic cetyltrimethylammonium bromide (CTAB). It provides good homeotropic alignment to 5CB molecules and links in a very stable way to the model protein BSA. Preliminary side measurements using DMOAP, which is widely used in NLC-based biosensors, did not give results comparable to those obtained with CTAB in terms of detection limit. This is most likely due to the more stable homeotropic NLC alignment produced by DMOAP with respect to CTAB [18]. Additional tests have been performed using a different protein, β-Lactoglobulin (BLG, Sigma Aldrich, St. Luis, MO, USA), the main whey protein in bovine milk. Compared to BSA, BLG is smaller, with a molecular weight of approximately 18.3 kDa and 162 amino acids [19]. BLG has an isoelectric point around 5.2, closer to the physiological pH with respect to that of BSA. For this reason, BLG exhibits a lower net negative charge.

To build the liquid crystal-based optical biosensors we confined 5CB between two 1.25 cm × 1.25 cm × 1mm microscope glass slides, separated by mylar stripes. We used 3 μm stripes for quantitative biosensing and 23 μm stripes for improving the detection limit. Before assembling the liquid crystal cells, the inner surfaces of the glass substrates were spin-coated with CTAB (see Supplementary Materials, Note S1). As a second step, one (for quantitative assay) or both (for improving the detection limit) glass substrates were coated by a solution of water and BSA (or BLG) at different concentrations ranging from 0 to 0.1 g/mL (Supplementary Materials, Note S2).

We investigated several strategies of protein deposition, such as (i) spin coating, following the same protocol used for CTAB surface functionalization described in the Supplementary Materials, Note S1; (ii) immersion of the substrate parallel to the protein solution/air interface at a constant speed of 7.5 × 10^−3^ mm/s followed by withdrawal at the same rate; and (iii) dip coating. Although all of them produced a detectable perturbation of the original homeotropic NLC orientation, the results were very different for each method. Specifically, in the case of spin coating of a drop of protein solution, the POM images were highly non-uniform, both in terms of intensity and color, making quantitative analysis unfeasible. Using top-down immersion, a better intensity distribution was observed compared to the spin coating technique; however, the images remained highly granular, with significant color variability. On the contrary, with dip coating we obtained highly uniform images in terms of color and pixel intensity, from which we could extract quantitative information on cell birefringence and on possible fluctuation of the optic axis. All the results presented in this study were obtained by depositing BSA and BLG on CTAB-coated glasses using the dip coating technique (details in Supplementary Material, Note S2).

Once assembled, the thickness of each cell was carefully measured by an interferometric technique. Then, cells were filled by capillarity with 5CB at T = 25 °C, corresponding to the nematic phase. During cell filling, the liquid crystal flow was kept parallel to the direction of dip coating.

A sketch of the steps required to build the proposed biosensors is reported in Figure 1b.

Liquid crystal cells were analyzed by polarizing optical microscopy and the presence of the protein molecules was detected through the color and brightness of the light transmitted by the POM analyzer. A 5× objective lens was used. Figure 1c shows a POM image of a cell with no protein, appearing completely dark regardless of the orientation of the cell with respect to the POM crossed polarizers, a signature of homeotropic alignment. These kinds of samples were used as reference cells. Each deviation from the dark images in Figure 1c is to be ascribed to the presence of a certain amount of BSA or BLG on one or both of the CTAB-coated glass surfaces. Python libraries (versions 3.13 and 3.14) were used for data processing (see Supplementary Materials, Note S3). Molecular dynamics simulations were also performed to investigate the interaction energies between the protein molecules and 5CB (see Supplementary Materials, Notes S4 and S5).

Additional experiments were carried out to evaluate the possibility of antigen–antibody binding on the functionalized glass surface, for a possible antibody-labeled detection. For this purpose, an assay was performed using a polyclonal antibody specific to BSA (Bovine Serum Albumin Polyclonal Antibody, Ab, from Thermo Fisher Scientific, Waltham, MA, USA). The choice of a polyclonal instead of monoclonal anti-BSA antibody was motivated by the unknown specific binding orientation of BSA on the CTAB-coated surface. Indeed, polyclonal antibodies recognize multiple epitopes on the BSA molecule, increasing the likelihood of binding. Glass substrates previously coated with CTAB were first functionalized with BSA at a concentration of 10^−7^ g/mL using the dip coating technique, followed by rinsing in ultrapure deionized water to remove unbound protein. The BSA concentration was selected to ensure the presence of a suitable amount of protein on the cell surface, without affecting too much the initial homeotropic alignment. In this way, the variation in brightness induced in the event of binding with the antibody was easily detectable. Subsequently, a second dip coating step was carried out by immersing the BSA-functionalized substrates in a polyclonal anti-BSA antibody solution (2 × 10^−6^ g/mL in 50 mM Tris-HCl buffer, pH 7.4), followed by overnight incubation at room temperature. After incubation, the substrates were removed from the solution (emergent dip coating) and rinsed again in ultrapure deionized water to eliminate any excess of antibody. A second CTAB-coated glass substrate with no BSA was used to assemble the cell. Cell thickness was fixed by 3 μm thick mylar spacers.

## 3. Results

The described protocol of protein deposition produces a coating of the cell substrate’s surface, which results in a perturbation of the liquid crystal alignment with respect to the homeotropic configuration obtained for c = 0 g/mL (Figure 1c). Such a perturbation extends over the whole field of view of the polarizing optical microscope and is therefore easily visualized and characterized.

**Figure 2:**
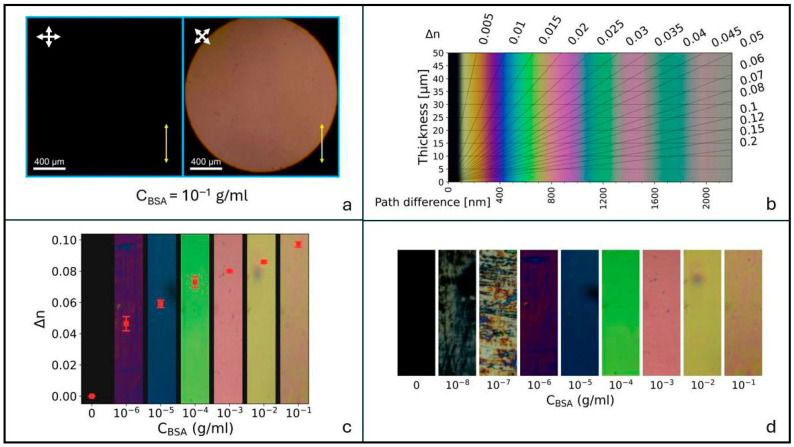
(**a**) POM image of a cell obtained by depositing 10^−1^ g/mL of BSA on one of the two confining surfaces coated with CTAB. The resulting alignment is hybrid. White crossed arrows represent the crossed POM polarizers. The yellow arrow indicates the direction of dip coating during BSA deposition (equal to the direction of 5CB flow during cell filling). (**b**) Custom-made Michel-Levy interference color chart, used to calculate the cell birefringence as described in the Supplementary Materials, Note S3. (**c**) Cells birefringence *Δn* vs. BSA concentration c_BSA_, for c_BSA_ ≥ 10^−6^ g/mL, superimposed to POM images of the cells obtained between crossed circular polarizers. Error bars were calculated based on the standard deviation of the optical retardance, determined by the Michel-Levy chart as described in the Supplementary Materials and on the experimental errors on cell thickness. (**d**) POM images of the cell appearance between crossed circular polarizers for all the concentrations producing a detectable signal (see text). The detection limit for these kinds of cells corresponds to 10^−8^ g/mL.

**Figure 3:**
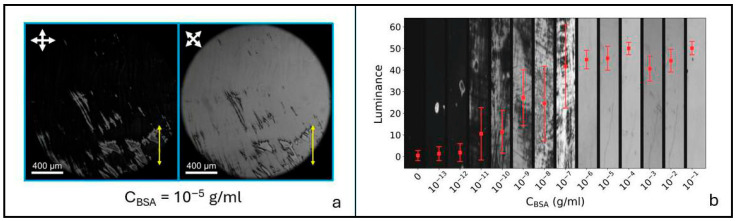
**(a)** POM image of a cell obtained by depositing 10^−5^ g/mL of BSA on both the confining surfaces coated with CTAB. The resulting alignment is homogeneous planar along the direction of dip coating, marked in yellow. White crossed arrows represent the crossed POM polarizers. (**b**) Cell luminance vs. BSA concentration. Error bars correspond to the standard deviation of the luminance, determined as described in the Supplementary Materials, Note S3. The detection limit corresponds to 10^−13^ g/mL.

Filling the cell while keeping the liquid crystal flow in the same direction as the dip coating allows us to obtain a well-defined alignment of the liquid crystal molecules over the whole cell. Specifically, cells where the protein was deposited on only one surface exhibit hybrid alignment (Figure 2), while those with BSA or BLG coating on both the confining surfaces acquire homogeneous planar alignment in the direction of the liquid crystal flow (Figure 3).

In the following we will report separately the experimental results obtained for the two kinds of cells and for the two proteins used as target analytes.

### 3.1. Single BSA Functionalization (Hybrid Cells)

LC cells where BSA was deposited on only one of the two CTAB-treated substrates have a measured thickness in the range 4–6 μm. These cells exhibit hybrid alignment (Figure 2).

For concentrations c_BSA_ ≥ 10^−6^ g/mL, BSA deposition results in a uniform cell texture over the area viewed between crossed polarizers. A typical example is reported in Figure 2a for c_BSA_ = 10^−1^ g/mL. The color of the image is a consequence of the birefringence *Δn* of the NLC cell, and when combined with the cell thickness *d*, it gives direct information on the value of this parameter. Indeed, when an NLC cell of thickness *d* is placed between the crossed linear polarizers of an optical microscope, the intensity *I* of the transmitted light is given by [20]

(1) $$I=I_{0\;}{sin}^{2}\frac{\delta }{2}{sin}^{2}2\varphi$$

In this expression, *I*_0_ is the intensity incident on the cell, *φ* is the angle between the direction of polarization of the incident light and the liquid crystal optic axis and *δ* is the phase shift experienced by the light as it travels through the liquid crystal cell. This latter arises due to the birefringence of liquid crystalline materials, namely the presence of two refractive indices, one for the light polarized orthogonal to the director (ordinary index *n_o_*), the other for light polarized along the director (extraordinary index, *n_e_*). The relationship between δ and the birefringence *Δn = n_e_ − n_o_* is formalized by the following expression:

(2) $$\delta =\frac{2\pi }{\lambda }d\Delta n$$

Here, the product *dΔn* represents the optical retardance experienced by the light wave. Depending on its value, the various components of the white light incident on the cell in the optical microscope undergo destructive or constructive interference, thus producing a colored transmitted light, with the specific color dependent on cell thickness and birefringence. If the thickness is known, as in our case, the color observed between crossed polarizers gives direct information on the cell birefringence. It is noteworthy that this is strictly true when the term sin^2^ 2*φ* in Equation (1) is equal to 1, that is, when the angle between the nematic director and the axis of the POM polarizer is π/4 or when circularly polarized light is used [20]. In these cases, the light transmitted by the POM analyzer only depends on the optical retardance and not on the direction of the cell optic axis.

To determine the birefringence from the color of POM images obtained between crossed polarizers, we used the custom-made Michel-Levy interference color chart reported in Figure 2b, which is based on the emission spectrum of the microscope light source used (details in the Supplementary Materials, Note S3). Cell birefringence was evaluated based on POM images obtained with circularly polarized light. This was realized using two circular polarizers, each one consisting of a broadband linear polarizer and a quarter-wave plate combined in a single polarizing element. Indeed, though the NLC alignment on the BSA-treated surface exhibits a well-defined easy axis, some fluctuation of the optic axis can be observed (see Supplementary Materials, Figure S2). This is ascribed to non-uniform areas in the BSA layer and might be responsible for experimental errors. The use of crossed circular polarizers allowed us to circumvent this problem.

In hybrid cells obtained by single BSA coating, the amount of perturbation to the homeotropic alignment depends on the protein concentration c_BSA_. Specifically, as c_BSA_ increases from 0 to 0.1 g/mL, the angle between the NLC optic axis and the direction of dip coating progressively decreases from π/2 (homeotropic alignment) to zero (planar alignment), due to the decrease in the anchoring energy of 5CB molecules on the CTAB layer induced by the presence of protein molecules and to the subsequent increase in the planar anchoring energy. This results in a variation in birefringence, so we expect a dependence of cell color on BSA concentration, which we indeed observe. This is reported in Figure 2c, where the birefringence evaluated with the aid of the custom-made Michel-Levy chart is plotted as a function of c_BSA_. The colored bands in the figure are POM images obtained between crossed circular polarizers. Each image corresponds to a different BSA concentration and to a different value of the birefringence.

Cell birefringence increases with BSA concentration up to 0.10 for c_BSA_ = 10^−1^ g/mL. Note that this value corresponds to about half the total birefringence of 5CB.

BSA concentrations lower than 10^−6^ g/mL give rise to non-uniform cell texture, where regions of zero birefringence alternate with colored, bright areas, indicating that BSA is not uniformly spread over the cell surface. In this case POM images do not allow for a reliable evaluation of the birefringence value. However, BSA sensing is still possible down to a concentration c_BSA_ = 10^−8^ g/mL (0.15 nM), as shown in Figure 2d.

### 3.2. Double BSA Functionalization (Planar Cells)

The sensitivity can be largely improved by coating BSA on both cell surfaces and increasing the cell thickness. This strategy produces an amplification of the detected signal (i.e., of the transmitted light due to the perturbation of NLC homeotropic alignment) based on the coupling of NLC molecules with the cell-confining surfaces. Indeed, as the cell thickness increases, the aligning effect of the CTAB-coated glass surfaces on the cell bulk weakens, thus resulting in a homeotropic alignment which is less stable than in thinner cells and thus more easily destabilized by BSA molecules even at low protein concentration. Moreover, coating both the confining surfaces with BSA contributes to enhancing the effect of protein adsorption on the biorecognition CTAB layer for an overall improvement of the detection limit.

When both the cell surfaces are coated with BSA, cells exhibit homogeneous planar alignment in the direction of dip coating, as shown in Figure 3a, where two POM images under crossed polarizers are reported for two different values of the angle *φ* between the NLC optic axis and the polarization of the light incident on the cell: *φ* = 0° (a’) and *φ* = 45° (a”). In agreement with Equation (1), the cell appears dark for *φ* = 0° and bright for *φ* = 45°, a condition where the maximum value of transmitted light is detected. The BSA concentration in Figure 3a is c_BSA_ = 10^−5^ g/mL.

Due to the larger thickness of these cells with respect to those described in the previous paragraph (nominal thickness *d* = 23 μm, corresponding to real thickness in the range 24–26 µm, as measured with interferometric techniques), POM images are not colored; therefore, the information on the birefringence is missing. Indeed, as cell thickness becomes large compared to the light wavelength, the order of the optical retardance increases and more wavelengths of light are removed from the spectrum, resulting in a color wash out. In the absence of colors, we characterized the capability of the biosensor to detect the presence of BSA on the biorecognition layer using the intensity of the transmitted light, as reported in Figure 3b. We extracted this parameter from POM images as the pixels’ average luminance, on a scale from 0 to 100 (see Supplementary Materials, Note S3). This quantity exhibits a first increase with BSA concentration for low values of c, due to the non-uniform spreading of BSA on the CTAB-treated surfaces. As the BSA layers become uniformly distributed, the luminance saturates, thus allowing in this case only qualitative sensing (i.e., protein present or protein absent, with no quantification of the amount). We highlight that this would be the case also for thin planar cells producing colored POM images. This happens because the alignment induced by BSA in the case of coating of both substrates is planar as soon as the protein becomes uniformly spread, which results in a uniform value of the birefringence, independent on BSA concentration.

The great advantage of using thicker cells with double BSA coating is the expected increase in the detection limit, visible in Figure 3b. In this case, the detection limit reaches the remarkable value of 10^−13^ g/mL (1.5 fM). It is noteworthy that this value corresponds to small local variations in the cell luminance, which are, however, highly reproducible, indicating the robustness of the obtained sensitivity.

### 3.3. Detection of Antibodies

For these experiments we used cells where only one of the two substrates was coated with BSA and kept the value of BSA concentration fixed at c_BSA_ = 10^−7^ g/mL. At this concentration, the perturbation induced by BSA on the original homeotropic alignment is small enough to guarantee a suitable contrast in the event of BSA/antibody binding. The antibody was dissolved in Tris-HCl, a solvent that does not interfere with the alignment provided by CTAB to the liquid crystal molecules, nor with the interaction between CTAB and BSA. These circumstances were demonstrated by tests on cells with one substrate dip-coated by BSA, at different concentrations ranging from c_BSA_ = 0 g/mL to c_BSA_ = 10^−5^ g/mL. Previous tests with the same antibody dissolved in Phosphate-Buffered Saline (PBS) showed that PBS disrupts the initial homeotropic alignment of the liquid crystal, resulting in a poor baseline for any kind of measurement.

**Figure 4:**
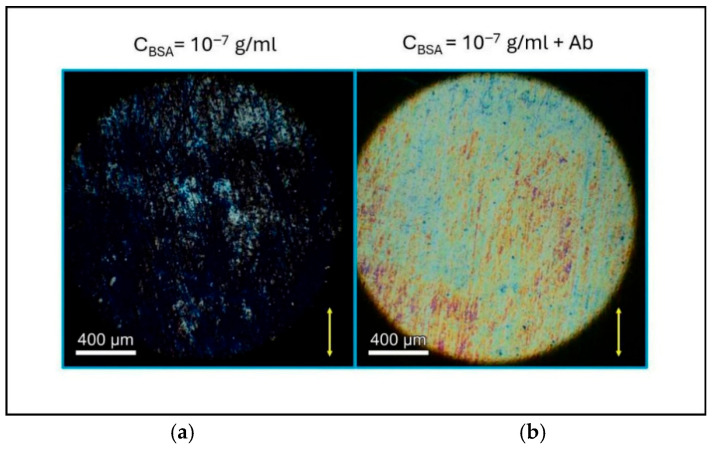
POM pictures of two hybrid cells with c_BSA_ = 10^−7^ g/mL, between crossed circular polarizers. The yellow arrow indicates the direction of dip coating during BSA deposition (equal to the direction of 5CB flow during cell filling). Images were obtained in the absence (**a**) and in the presence (**b**) of the unconjugated polyclonal anti-BSA antibody deposited as described in the text. The different appearance of the cells allows for antibody detection.

Figure 4 shows two POM images obtained with crossed circular polarizers of a hybrid cell of thickness *d* = 5 µm. On the left-hand side, no antibody has been added to the cell, while on the right-hand side the polyclonal anti-BSA antibody has been dip-coated on the BSA-treated surface. The difference in brightness between the two images is evident, demonstrating the possibility of using the proposed NLC-based biosensor for the detection of antibodies and possibly of protein/antibody interaction.

### 3.4. Experiments with BLG

**Figure 5:**
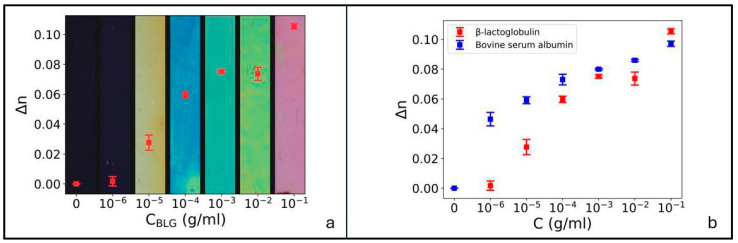
(**a**) Cells birefringence *Δn* vs. BLG concentration c_BLG_, for c_BLG_ ≥ 10^−6^ g/mL, superimposed to POM images of the cells between crossed circular polarizers; (**b**) comparison between the curves *Δn* vs. c obtained for the two proteins used in this study. The difference between the two suggests a certain degree of specificity of the proposed biosensor.

**Figure 6:**
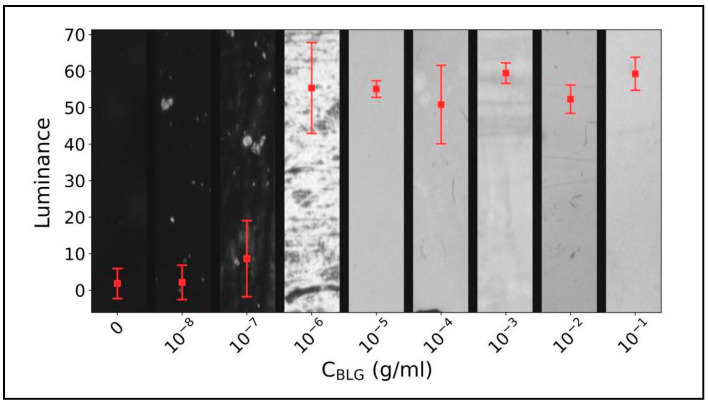
Cells luminance vs. BLG concentration, c_BLG_. The detection limit is lower by at least four orders of magnitude with respect to that obtained with BSA.

To further investigate the capabilities of the proposed system, we performed additional experiments with BLG, a protein different from BSA, smaller in size and with a weaker negative charge. The protocol of deposition of BLG and of cell preparation is the same as that described for BSA. We realized both hybrid cells, obtained by coating only one confining surface, and planar cells, with two substrates coated with the protein to detect. Results are reported in Figure 5a (hybrid cells) and Figure 6 (planar cells).

The birefringence in Figure 5a exhibits the same increasing trend as in Figure 2, thus allowing for quantitative sensing based on the color of POM images. The direct comparison between the two graphs, reported in Figure 5b, shows that the values of *Δn* for the same protein concentration are different for BLG and BSA, indicating that the biosensor proposed in this work reacts in a selective way to the specific protein it detects, which might be a promising path toward the realization of specific biosensors. Specifically, the birefringence curve is expected to depend on the properties of the tested protein, namely its structure, size, charge, and the type of residue. This is also suggested by molecular dynamics simulations reported in Supplementary Materials showing that 5CB interacts very well with BSA aromatic amino acids, better than in case of BLG. Indeed, BSA presents several binding pockets containing aromatic amino acids, which allow for π–π stacking interactions with the biphenyl groups of 5CB. In contrast, although the percentage of aromatic residues in BLG is very similar to that of BSA, only one stable binding pocket was found for the liquid crystal molecule (see Supplementary Materials, Note S5). Note that 5CB also contains a cyano group, which is a hydrogen bond acceptor; however, although hydrogen bonds were observed during the simulations, the interactions appear to be mainly driven by quadrupolar interactions between aromatic rings.

Figure 6 reports the cell luminance as a function of BLG concentration, for thick planar cells. The figure shows a detection limit of 10^−8^ g/mL (0.54 nM), that is at least four orders of magnitude worse with respect to the one obtained in the case of BSA. We understand this behavior as due to a different strength of interaction between the two proteins and CTAB, as also suggested by the different values of c corresponding to uniform protein coating of the CTAB layer (c_BLG_ = 10^−5^ g/mL vs. c_BSA_ = 10^−6^ g/mL). Although an analysis of the interaction between proteins and the sensing layer is beyond the scope of this manuscript, the negative charge of both BLG and BSA suggests at least a certain amount of electrostatic interaction with the positively charged quaternary ammonium group of CTAB. As BSA is more negatively charged than BLG, this could explain why the former interacts stronger than the latter. Molecular dynamic simulation indeed revealed strong electrostatic interactions with negatively charged amino acid residues, particularly glutamic acid. Once again, the interaction energies are more favorable with BSA and less significant with BLG (see Supplementary Materials, Notes S4 and S5).

Interestingly, tests with the polyclonal anti-BSA antibody, such as those described in the previous section, did not give any results in the case of BLG. Although this was expected, these tests demonstrated that the optical response of the liquid crystal observed in Figure 4 is uniquely attributable to the specific interaction between BSA and its corresponding antibody, rather than to non-specific protein–antibody interactions.

## 4. Conclusions

We reported on an optical biosensor based on nematic liquid crystals for the quantitative and highly sensitive detection of proteins. Although liquid crystal-based biosensors have been established for several years, we proposed a specific protocol of cell preparation that allows for a quantitative evaluation of protein concentration and a significant improvement of the detection limit with respect to values reported in the literature for these kinds of systems. The biosensor proposed in this manuscript exhibits a certain degree of specificity, rarely found in liquid crystals biosensors, that can be further analyzed in the future by testing additional proteins. Moreover, it can be used for the labeled detection of antibodies and, possibly, for the characterization of their interaction with BSA or other proteins. All these features are combined in a very simple and inexpensive system, characterized by a visual detection method affordable also for non-experts.

One of the key points of this work is the functionalization of the CTAB-coated surfaces, which we carried out using the dip coating technique. This approach offers several advantages: enhanced homogeneity of the protein layer, improved reproducibility between samples, and the possibility to prepare multiple substrates simultaneously under identical conditions, ultimately improving the stability and predictability of the liquid crystal alignment. This procedure combined with (i) the use of an aligning agent that acts as a proper biorecognition element and assures good initial homeotropic alignment while exhibiting not too strong interaction with LC molecules; (ii) the proper choice of cell thicknesses; and (iii) the preparation of both hybrid and homogeneous planar cells, allows obtaining quantitative sensing and an extremely high detection limit.

Notably, the procedure we used to obtain signal amplification and quantitative sensing of the target analyte does not require externally applied fields or cumbersome measurements of the detected signal.

## Data Availability

The original contributions presented in this study are included in the article/Supplementary Material. Further inquiries can be directed to the corresponding authors.

## Supplementary Materials

The following supporting information can be downloaded at: https://www.mdpi.com/article/10.3390/bios16030168/s1, Figure S1: CIELAB color space converted to sRGB for visualization. Since *Lab* is a perceptually uniform, device-independent color space, it cannot be directly displayed on screen; it must first be mapped to a device-dependent space like RGB, which introduces gamut limitations and perceptual distortions for out-of-range colors. Figure S2: Representation using the jet colormap of the fluctuations of the optical axes (expressed in radians) in a cell with hybrid alignment. (A) Experimental image obtained with crossed linear polarizers with the polarizer (P) at 0° with respect to the cell filling direction, and the corresponding mapping of the optical axes. (B) Experimental image obtained with crossed linear polarizers with the polarizer (P) at 45° with respect to the cell filling direction, and the corresponding mapping of the optical axes. The color bar indicates the fluctuation angle in radians. Figure S3: Sequence of circularly polarized images from the wedge cell used to generate the custom Michel-Levy chart. Figure S4: Illustrative example of birefringence extraction by matching the circularly polarized image with the custom Michel-Levy chart. (A) POM image between crossed circular polarizers; (B) reconstructed image using the pixels from the Michel-Levy chart that best matched the pixels of the experimental image; (C) construction of the Gaussian fit of the pixel match along the x-axis, representing the optical retardation. Figure S5: RMSD data for each protein–ligand pair subjected to simulation (one binding site per pair is represented). BSA–5CB test (A) and BSA–CTAB test (C). As observed, after an initial relaxation of the ligand within the binding pocket—indicated by a slight increase in RMSD—the value remains stable throughout the simulation. The same tests for BLG–5CB and BLG–CTAB are reported in (B) and (D), respectively. In these cases, the RMSD data clearly indicates that the ligand exits the initial binding pocket. Figure S6: (A) Distance between an aromatic ring of 5CB and a pyrrole ring of a tryptophan residue (TRP213) as a function of simulation time. The red line indicates the cut-off value (0.4 nm) below which π–π stacking interactions are considered significant. (B) Graphical representation of a simulation frame using ChimeraX, highlighting the π–π stacking interaction described in the plot [21,22,23,24,25,26,27]. Note S1: Preparation of CTAB-coated substrates; Note S2: Protein deposition; Note S3: Data analysis; Note S4: Molecular docking; Note S5: Molecular Dynamics Simulations.
