# Supplementary material for: Liquid Crystal-Based Optical Biosensor for Quantitative, Highly Sensitive Detection of Proteins
Source: Biosensors (Basel). 2026 Mar 17;16(3):168. doi: 10.3390/bios16030168 (PMC13023930; doi:10.3390/bios16030168)
Supplement: Supplementary file 1 [file biosensors-16-00168-s001.zip › biosensors-4190616-supplementary.pdf]

Supplementary Information

Note S1: Preparation of CTAB-coated substrates

Cationic cetyltrimethylammonium bromide (CTAB) was deposited by spin coating on glass slides previously treated with the UV ozone cleaner for 40 minutes. 30 µl of CTAB diluted in isopropanol (0,1% w/v) was put on the glass surface and spin coated at 2000 RPM for 10 seconds, followed by a gradual decrease to 1000 RPM over the next 40 seconds and then by a short final run at 1000 RPM for 10 seconds to stabilize the film. Finally, the slides were baked for 30 minutes at 120 °C, then gradually cooled down for BSA deposition.

Note S2: Protein deposition

To achieve a uniform and reproducible BSA coating on the CTAB coated glass surface, the dip coating technique was employed. This method involves controlled immersion and withdrawal of the substrate from the protein solution. Each glass slide was firmly secured using a clamp connected to a micrometric translation stage with the Newport 850G actuator operated by a Newport ESP300 controller. The immersion was carried out at a constant velocity of $7.5x10^{-3}$ mm/s, with a total immersion time of 15 minutes, followed by a 15-minute withdrawal at the same speed. This slow, controlled movement ensures a homogeneous protein deposition across the entire surface.

After the dip-coating process, the slides were rinsed in ultra-pure deionized water at the same withdrawal speed to remove any unbound protein. The same protocol was used for BLG deposition.

Once assembled as described in the main text, the biosensors have been filled by capillarity with 5CB in the nematic phase. In doing this, the direction of 5CB flow was carefully matched to the direction of dip coating (i.e. the withdrawal direction) of the protein solutions.

Note S3: Data analysis

Data processing and analysis were carried out using custom Python scripts and other open-source libraries [1, 2] to extract quantitative information from the experimental measurements. These tools enabled the quantification of optical axis fluctuations from polarized light microscopy images, and the calculation of birefringence and luminance values essential for characterizing the optical behavior of the liquid crystal cells.

Fluctuations of the optical axis

Each polarized light optical micrograph (POM) image was read as three-dimensional *NumPy* array [1] where the first two axes represent the respective height and width of the image in terms of pixels. Along the third axis there are three values that describe intensity and color of the pixels, according to the *Lab* (CIELAB) color space. *L* (i.e the first value) describes the luminance, ranging from 0 (black) to 100 (white). This value controls how bright or dark a color appears. The *a* component (second value) represents the green-to-red scale, so a negative value indicates a pixel tending towards green, while a positive value indicates a pixel tending towards red. Finally, the *b* component (third value) represents the blue-to-yellow scale, so a negative value indicates a pixel tending towards blue and a positive value indicates a pixel tending towards yellow.

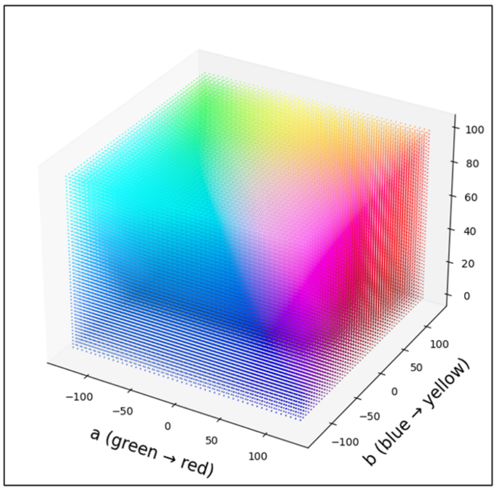

Figure S1: CIELAB color space converted to sRGB for visualization. Since *Lab* is a perceptually uniform, device-independent color space, it cannot be directly displayed on screen; it must first be mapped to a device-dependent space like RGB, which introduces gamut limitations and perceptual distortions for out-of-range colors.

For the calculation of the optical axis fluctuations, the sample images obtained at 0° and 45° with linear crossed polarizers were imported as an RGB image with utilities provided by the *Scikit-image* library [2]. Subsequently, each pixel of the image was converted to the *Lab* format using the “Colour” library (an open-source Python library for color science) [3] and an array relative to the optical axis fluctuations was extracted using equation (1) of the main text, with the value of luminance as intensity. The correctness and consistency of the recovery of the optical axis was validated using perfectly planar reference cells.

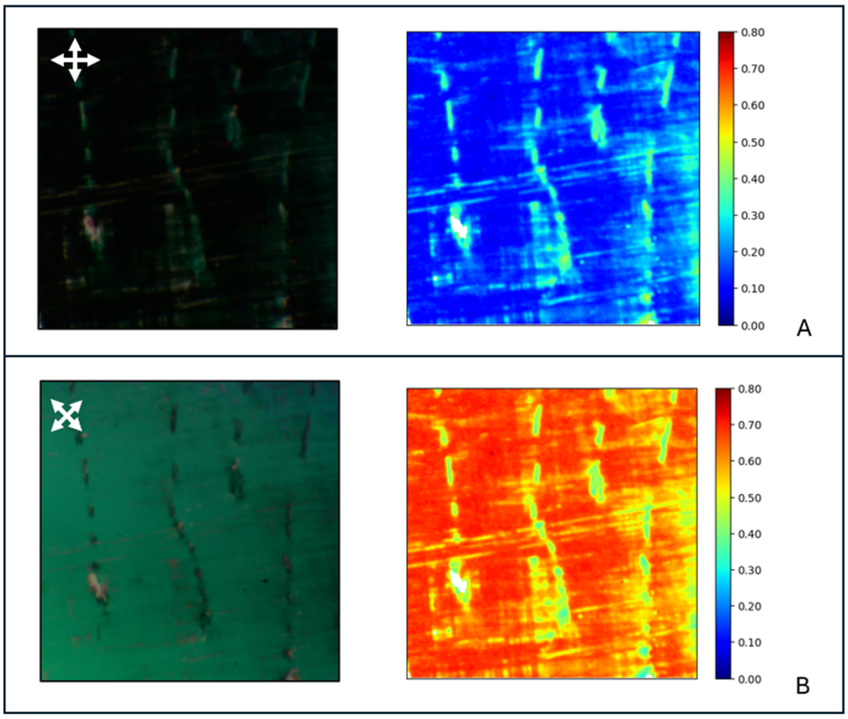

Figure S2: Representation using the jet colormap of the fluctuations of the optical axes (expressed in radians) in a cell with hybrid alignment. a) Experimental image obtained with crossed linear polarizers with the polarizer (P) at 0° with respect to the cell filling direction, and the corresponding mapping of the optical axes. b) Experimental image obtained with crossed linear polarizers with the polarizer (P) at 45° with respect to the cell filling direction, and the corresponding mapping of the optical axes. The color bar indicates the fluctuation angle in radians

Custom Michel-Levy chart and birefringence extraction

The colors of the POM images depend both on material properties (refractive indices and their dispersion) and on the spectral properties of the illumination system. Therefore, great care must be taken to extract accurate information about the birefringence. To this purpose, a customized version of the Michel-Levy interference color chart suitable for birefringence quantification was built, which properly accounts for the experimental conditions. The method relied on a sequential acquisition of POM images along a wedge cell filled with the same nematic liquid crystal used in the experiments (5CB), under crossed circular polarizers and fixed operating conditions of the microscope (illumination, aperture diaphragm and so on). In this configuration, optical retardation varies progressively along one direction of the wedge cell, allowing for a continuous gradient of birefringence values across the field of view.

A series of circularly polarized images were acquired by shifting the wedge cell laterally under the microscope. These images were saved and later processed in batch. The reconstruction code iterates through the image files stored in a designated folder and loads each one as an RGB array. To facilitate the reconstruction, selected regions of interest from each image (chosen for their uniformity and clarity) were cropped and concatenated horizontally, producing a single stitched image that represents the retardation gradient over a wider spatial range.

This reconstructed image was then subjected to pixel level smoothing to reduce discontinuities and local non uniformities caused by small but perceptible illumination fluctuations or sample imperfections. The result is a custom Michel-Levy chart calibrated to the specific light source (halogen lamp, intensity and spectrum) and to the spectral response of the components along optical path of the setup. Each pixel was mapped to a known optical retardation value expressed in nanometers, allowing for direct birefringence quantification from microscopy images.

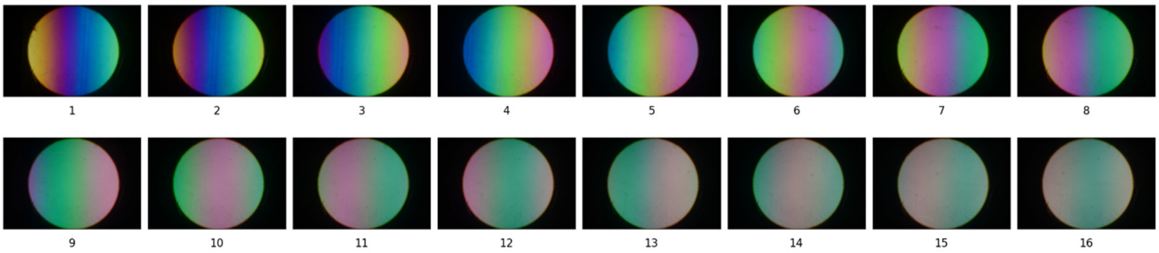

Figure S3: Sequence of circularly polarized images from the wedge cell used to generate the custom Michel-Levy chart.

To extract birefringence data, images acquired under circularly crossed polarizers were used. Each image was first imported and converted into the CIELAB color space, which separates luminance (*L*) from chromatic components (*a* and *b*), allowing for accurate color comparison. For each pixel in the experimental image, the corresponding birefringence value was determined by comparing its Lab values with those of the custom Michel-Levy chart.

The color matching process was performed using the CIE2000 function from the Colour library. This function implements the CIEDE2000 formula, which evaluates perceptual color differences by considering not only luminance and chromatic shifts (hue and chroma), but also including specific correction terms to account for perceptual non-linearity and improve accuracy for small color differences. The output of the function is a scalar value (ΔE_{00}) that quantifies the color difference between two pixels. A lower ΔE_{00} indicates a better match, with values close to one representing the threshold for human perceptibility.

The index of the pixel on the Michel-Levy chart that yielded the minimum ΔE_{00} was then used to determine the optical retardation. Finally, by combining the retardation value with the thickness measured previously by interferometric analysis, the birefringence was computed pixel by pixel across the image.

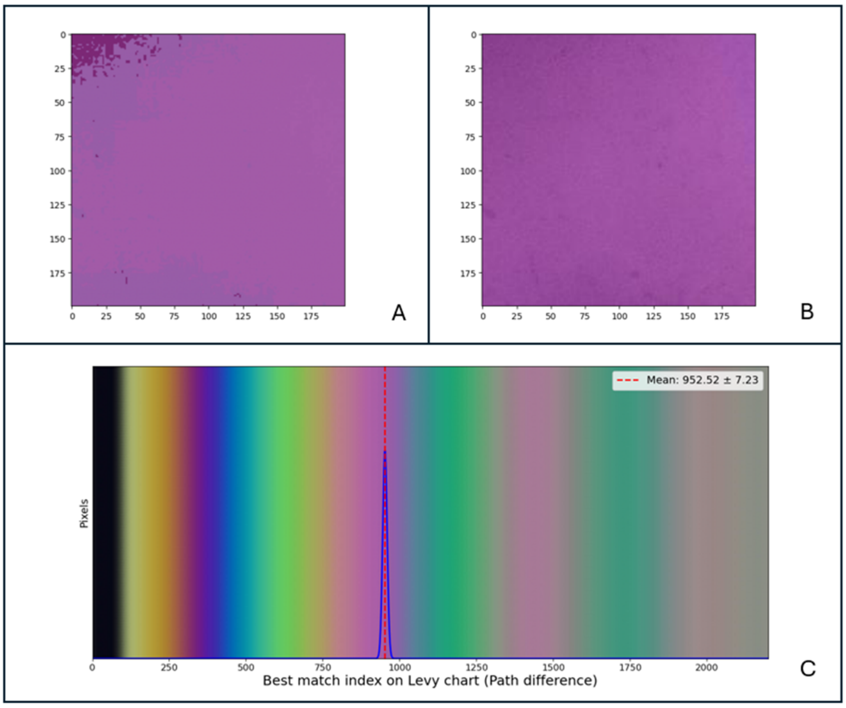

Figure S4: Illustrative example of birefringence extraction by matching the circularly polarized image with the custom Michel-Levy chart. a) POM image between crossed circular polarizers; b) Reconstructed image using the pixels from the Michel-Levy chart that best matched the pixels of the experimental image; c) Construction of the Gaussian fit of the pixel match along the x-axis, representing the optical retardation.

Luminance calculation

The luminance of polarized optical images is highly dependent on the orientation of the liquid crystal molecules with respect to the polarization direction of the incident light. This orientation is influenced by surface interactions at the LC/substrate interface. In the present study, luminance values were extracted and analyzed to investigate the effect of surface functionalization with BSA and BLG alone, and with BSA followed by antibody immobilization.

Quantitative comparison of luminance distributions among different surface conditions enables an indirect evaluation of how biochemical modifications influence the optical behavior of nematic LCs, thereby serving as a sensitive tool for label-free bio-detection.

To quantitatively analyze the luminance variations observed in polarized optical images, the original RGB images were converted to the *Lab* color space. This color space separates luminance (*L*) from chromatic components (*a* and *b*). The luminance channel was extracted and averaged to obtain a representative measure of light intensity across the sample.

Note S4: Molecular docking

Molecular docking studies were performed to investigate the sites and energies of interaction between the proteins used in this study (BSA and BLG) and the ligands 5CB and CTAB, using AutoDock software (version 4.2.6) [4]. As starting PDB files, i.e., the files containing the coordinates of the protein atoms,

3v03 was used for BSA, while 2q2m was used for BLG. The ligands were built using ChimeraX software [5]. In all cases, the protein was treated as rigid, and partial charges were assigned using the Kollmann model, while the ligands were considered flexible and partial charges were assigned according to the Gasteiger model. Molecular docking was performed using the Lamarckian Genetic Algorithm with 100 runs to ensure adequate conformational sampling and reliable identification of binding poses. After the calculation, the best pose from the best cluster for each grid box was selected. This process was repeated for all the grid boxes. All the selected poses were optimized with additional docking, centering the grid box on the ligand. To analyze the entire surface of the proteins, 5 grid boxes were used for BSA and 3 for BLG. Accordingly, 5 poses for each ligand (CTAB and 5CB) were selected for BSA, and 3 poses for each ligand were selected for BLG, to be submitted to molecular dynamics simulations.

Note S5: Molecular Dynamics Simulations

Molecular dynamics simulations were performed using GROMACS software (version 2024.5) [6] and the CHARMM36 forcefield. For each protein–ligand pair, the simulations were based on the best pose from the best cluster selected for each grid box during the docking step, as previously described. Only those binding sites were considered for simulation. Each simulation was run for 100 ns, a sufficient time to consider a binding site as stable. For the simulation analysis, several data were extracted as a function of simulation time to confirm the quality of the binding sites for both ligands. Interaction analyses were carried out using the Python library PLIP [7] for every frame of the simulation trajectories, specifically to identify and monitor hydrogen bonds, π–π stacking interactions, and electrostatic interactions over time. In addition, RMSD (Root Mean Square Deviation) values of the ligand relative to the aligned protein were calculated to assess the stability of the ligand within the binding site throughout the simulation.

For all the simulations involving BSA, the RMSD values were found to be acceptable with both CTAB and 5CB, confirming the stability and validity of all predicted binding sites. In contrast, for BLG, only one binding site proved to be stable for each ligand, with one valid site identified for CTAB and one for 5CB. In the remaining sites, the ligand consistently exited the binding pocket during the simulation.

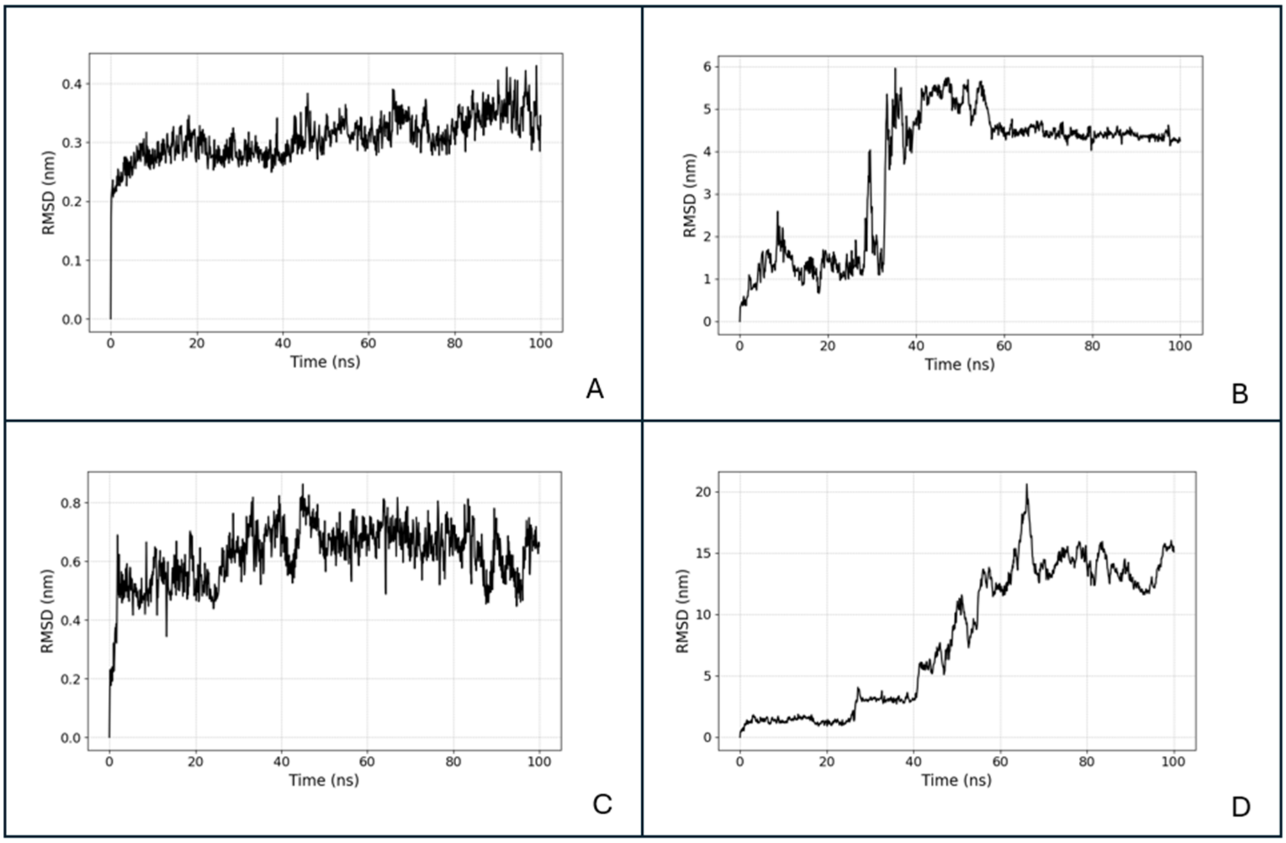

Figure S5: RMSD data for each protein–ligand pair subjected to simulation (one binding site per pair is represented). BSA–5CB test (A) and BSA–CTAB test (C). As observed, after an initial relaxation of the ligand within the binding pocket—indicated by a slight increase in RMSD—the value remains stable throughout the simulation. The same tests for BLG–5CB and BLG–CTAB are reported in B and D, respectively. In these cases, the RMSD data clearly indicates that the ligand exits the initial binding pocket.

As for the interactions identified with PLIP in the case of 5CB, hydrogen bonds were found mainly due to its cyano group, which is a good acceptor. Additionally, potential π–π stacking interactions arise from the two biphenyl rings of 5CB. The results show that 5CB forms hydrogen bonds and π–π stacking interactions with both proteins; however, with BSA the interaction is much more stable, as at least one π–π stacking interaction persists throughout the entire simulation in every binding site tested, stabilizing the complex. These π–π interactions occur with aromatic amino acids such as phenylalanine, tyrosine, and tryptophan. In the case of BLG, this phenomenon is observed in only one tested site. Moreover, hydrogen bonds were found to be stable in only a few of the tested sites.

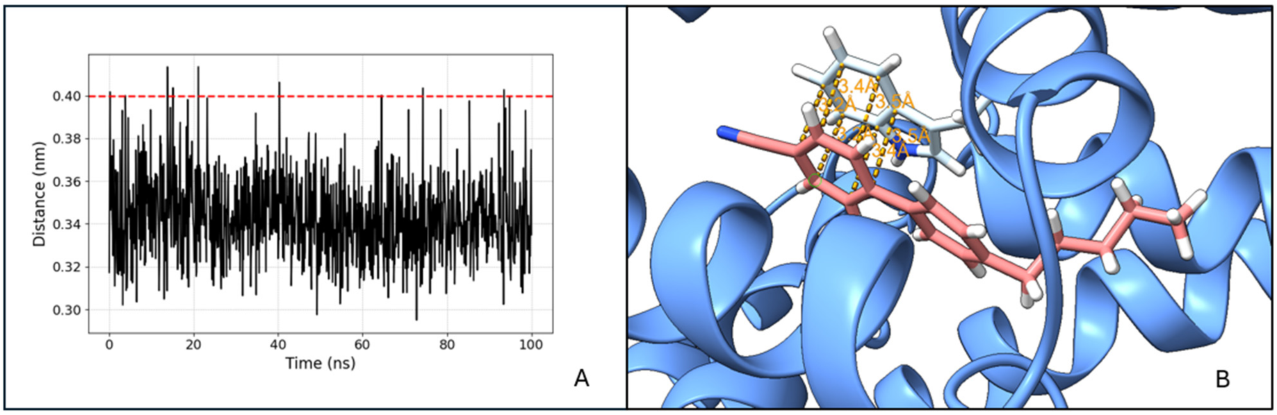

Figure S6: a) Distance between an aromatic ring of 5CB and a pyrrole ring of a tryptophan residue (TRP213) as a function of simulation time. The red line indicates the cut-off value (0.4 nm) below which π–π stacking interactions are considered significant. b) Graphical representation of a simulation frame using ChimeraX, highlighting the π–π stacking interaction described in the plot.

The PLIP analysis performed with CTAB indicates electrostatic interactions with negatively charged amino acids such as glutamic acid and aspartic acid in every tested binding site for both BLG and BSA. However, for BSA, each binding pocket features a stable electrostatic interaction that persists throughout the simulation time, whereas for BLG only a single stable site was observed.

References:

[1] C.R. Harris, K.J. Millman, S.J. van der Walt et al. *Array programming with NumPy*. Nature **585**, 357–362 (2020).

[2] S. Van der Walt et al. (2014), *scikit-image: image processing in Python*. PeerJ **2,** e453 (2014).

[3] T. Mansencal et al. *Colour*, 0.4.1; Zenodo: Geneva, Switzerland, 2022.

[4] S. Forli, R. Huey, M.E. Pique, M.F. Sanner, D.S. Goodsell, A.J. Olson. Computational protein–ligand docking and virtual drug screening with the AutoDock suite. *Nat. Protoc.* 11, 905–919 (2016).

[5] E.F. Pettersen, T.D. Goddard, C.C. Huang, E.C. Meng, G.S. Couch, T.I. Croll, J.H. Morris, T.E. Ferrin. UCSF ChimeraX: Structure visualization for researchers, educators, and developers. *Protein Sci.* 30(1), 70–82 (2021).

[6] GROMACS Development Team. GROMACS 2024.5; Zenodo: 2024.

[7] M.F. Adasme, K.L. Linnemann, S. Bolz, F. Kaiser, S. Salentin, V.J. Haupt, M. Schroeder. PLIP 2021: Expanding the scope of the protein–ligand interaction profiler to DNA and RNA. *Nucleic Acids Res.* 49(W1), W530–W534 (2021).
